# Patient-Controlled Analgesia (PCA): Intravenous Administration (IV-PCA) versus Oral Administration (Oral-PCA) by Using a Novel Device (PCoA® Acute) for Hospitalized Patients with Acute Postoperative Pain—A Comparative Retrospective Study

**DOI:** 10.1155/2021/2542010

**Published:** 2021-05-04

**Authors:** Stefan Wirz, Stefanie Seidensticker, Ronit Shtrichman

**Affiliations:** ^1^Department of Anesthesiology, Interdisciplinary Intensive Care, Pain Medicine/Palliative Medicine, Center for Pain Medicine, Center for Weaning, GFO-Clinics Bonn/CURA Hospital, Bad Honnef, Germany; ^2^Dosentrx Ltd., 2 Hamelacha Street, Har Tuv Industrial Zone A, Beit-Shemesh 9980101, Israel

## Abstract

**Background:**

Acute postoperative pain delays recovery and increases morbidity and mortality. Opioid therapy is effective but is accompanied by adverse reactions. Patient-controlled analgesia (PCA) enables self-administration of analgesics. Oral-PCA is a safe and beneficial alternative to intravenous (IV) PCA. We have developed a novel Oral-PCA device, which enables self-administration of solid pills to the patient's mouth. This is a retrospective study comparing the effectiveness and usability of this novel Oral-PCA with those of IV-PCA.

**Methods:**

Medical records of patients who received PCA following gynecology and orthopedic surgeries were analyzed. The control cohort (*n* = 61) received oxycodone by IV-PCA. The test cohort (*n* = 44) received oxycodone by Oral-PCA via the PCoA Acute device. Outcome measures include the Numeric Rating Scale (NRS) score at rest and movement, side effects, technical difficulties, bolus dose administered, and bolus dose requested.

**Results:**

Patient demographics, initial NRS, and PCA duration were comparable between cohorts. NRS reduction in rest and movement was stronger in the Oral-PCA cohort (rest: 1.61 and 2.27, *P* = 0.077; movement: 2.05 and 2.84, *P* = 0.039), indicating better pain control and mobility for Oral-PCA. Side effect rates were comparable between cohorts (9% and 11% of patients who experienced side effects, *P* = 1.000). The rate of technological difficulties was higher in the Oral-PCoA cohort (19.7% and 36.4%, *P* = 0.056). The mean total bolus dose administered to patients was comparable in both cohorts (18.32 mg and 21.14 mg oxycodone, *P* = 0.270). However, the mean total boluses requested by patients during lockout intervals were lower in the Oral-PCA cohort (12.8 mg and 6.82 mg oxycodone, *P* = 0.004), indicating better pain control.

**Conclusions:**

Oral-PCA by using PCoA® Acute provides pain control and usability which is noninferior to the IV-PCA, as well as superior to pain reduction in rest and movement. These results, along with the noninvasiveness, medication flexibility, and reduced cost, suggest the potential of Oral-PCA, by using PCoA Acute, to replace IV-PCA for postoperative analgesia.

## 1. Introduction

Acute pain is the most common symptom after surgery. Up to 93.7% of patients in all surgical wards suffer from acute pain. It is experienced immediately and up to 7 days after surgery and is considered chronic when lasting over 3 months following the surgery. Evidence suggests that almost half of the patients report inadequate postoperative pain relief, with high level of dissatisfaction with their pain management [1]. Subjective, insufficient assessment of pain is another reason for suboptimal acute pain management [2].

There are numerous short- and long-term consequences of inadequately treated acute pain, including immobilization, higher rates of pneumonia, sympathetic activation leading to a higher oxygen consumption, hyperglycemia, insulin resistance, an increased risk of infection, and decreased patient comfort and satisfaction. Moreover, it can lead to chronic, persistent pain, which may reveal psychosocial impairments as well, lowering return to work rates and being associated with depression or anxiety [3]. In contrast, adequate treatment of acute postoperative pain can improve the patient quality of life and satisfaction with care, enhance early mobilization, and reduce hospital stay and cost of care. Yet, acute pain remains undertreated and its management is suboptimal [4].

Nurses play a key role in the assessment and management of patients' pain. However, nurses tend to underestimate patients' pain and undermedicate patients for their pain [5]. The mainstay of postoperative pain therapy is opioids, but opioids have significant side effects, such as respiratory depression, nausea, and vomiting, and their long-term use can lead to dependence and addiction [6]. Consequently, nurse provision of analgesics to the patient is a strictly controlled and time-consuming procedure, posing significant burden on the nursing staff [7]. The solution is to allow patients to self-control their pain by self-administering their pain medication as needed [8].

The patient-controlled analgesia (PCA) technique is a validated and frequently used delivery method providing self-administered and predetermined doses of analgesic medication to relieve acute pain. PCA can minimize the occurrence of gaps in analgesic administration, providing more uniform analgesia and eliminating painful waiting periods between requesting and receiving the medication. Patients who received PCA experienced less acute pain and had a shorter hospital stay. PCA has become a standard of care in postoperative acute pain management in the hospital setting as it can provide better pain control and greater patient satisfaction [9].

Different modalities of PCA have been developed. The most common PCA routes are the intravenous (IV) PCA and epidural PCA. However, these PCA modalities are invasive, and they restrict patient mobility and require extensive staff time and resources. In addition, IV-PCA is prone to human dosing errors as well as mechanical malfunctions, causing harm to patients and adding a significant cost burden to the healthcare systems [10].

Cost analysis of a 72-hour treatment of postoperative pain in patients undergoing major surgical procedures had shown that IV-PCA is the most expensive treatment (€245.66/patient), requiring massive involvement of the clinical team, whereas the oral analgesic is associated with the lowest cost (€6.25/patient). This explains the relatively low uptake of the IV-PCA treatment modality, despite it being included in many treatment guidelines [11].

Noninvasive PCA may overcome the shortcomings of IV-PCA. Several systems have been developed and evaluated for their efficacy and safety compared to IV-PCA [12].

Sublingual PCA: sufentanil sublingual tablet system (SSTS, Zalviso®) is a preprogrammed, noninvasive, handheld device which dispenses a single sufentanil bioadhesive nanotablet, with a 20-minute lockout period. Clinical studies evaluating SSTS demonstrated noninferior pain relief and better ease of care, satisfaction, and mobility compared to IV-morphine PCA. Adverse events were comparable between both modalities [13]. The main limitations are the high cost and inability of this system to be used for medications other than sufentanil.

Transdermal PCA: fentanyl iontophoretic transdermal system (FITS, IONSYS®) is a noninvasive, preprogrammed, patient-activated drug delivery system. The system is applied onto the intact skin of the patient. Each activation of the FITS delivers a fixed fentanyl dose, with a 10-minute lockout between doses. Limitations are the need to remove and discard the FITS before clinical procedures are initiated and the limitation to only one drug-fentanyl. Studies identified better mobility with FITS but higher early discontinuation rate and inadequate analgesic treatment compared with the IV-morphine PCA [14].

Oral-PCA: an adaptive dispenser is required for the management of Oral-PCA using tablets that are to be swallowed. Medication provision is carried out according to a preprogrammed treatment plan. This modality is flexible and enables the use of various oral analgesics in the tablet form. Patients eligible for this technique should be cognitively able to handle an electronic device and to independently manage the delivery of the tablets. Patients using the Oral-PCA route benefit from the advantages of self-controlled pain treatment, while having lower risk of infection and better postoperative mobility. The clinical team also benefits from reduced workload and lower cost of treatment. Therefore, the Oral-PCA modality is strongly recommended [15]. Several Oral-PCA devices have been evaluated in recent years and found to be safe and effective for provision of postoperative pain medication in the clinical setting [16, 17].

**Figure 1:**
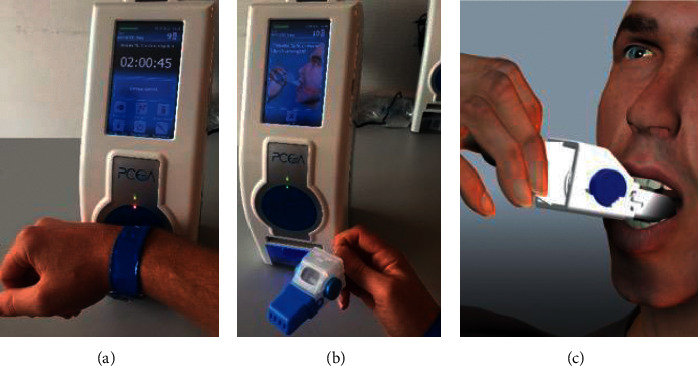
PCoA® Acute: the device is set up and positioned near the patient's bedside. (a) Patient identity is confirmed by registration of the RFID wristband. (b) The patient withdraws the pillbox to take a pill. (c) The patient applies light suction on the pillbox mouthpiece to receive a pill.

PCoA® Acute (Dosentrx®) is a novel preprogrammed, personalized, noninvasive Oral-PCA system (Figure 1). The system supports any oral pain medication in a pill or capsule form and easily integrates into the clinic routine. It safely delivers and tracks each pill from the device to the patient's mouth and thus verifies and records the consumption of each pill by the patient. PCoA® Acute dispenses to the prescribed patient only, using RFID identification. It has a touchscreen which allows the patient to self-report their pain level and side effects. A cloud-based portal enables remote monitoring, generation of alerts to the clinical team, and data management.

The PCoA® Acute system comprises the following components: (1) a drug dispensing unit: a safe storage container which dispenses the medication to the patient according to a preprogrammed regimen; (2) a pillbox: a patient-specific, mouth-actuated, disposable receptacle, from which the patient receives the pills. The pillbox releases a pill only when subject to negative sucking pressure, which delivers the pill onto the patient's tongue. This technology allows stringent control over the analgesic drugs consumed by the patient.

A feasibility study evaluated PCoA® Acute's safety, functionality, and usability [18]; the study compared patients who received oral analgesics in two different modes: (a) manual delivery of oral opioids by nursing staff and (b) Oral-PCA by using PCoA® Acute. The major findings were that there were no incidences of severe adverse events; the average pill intake time significantly reduced; PCoA® Acute was effective in pain reduction, and over 90% of the patients were satisfied with its use. This study therefore confirmed that PCoA® Acute is safe and effective, that its use can optimize the process of postoperative pain management, and that it is well accepted by patients and medical staff alike.

PCoA® Acute was deployed in our clinic for the treatment of postoperative acute pain by Oral-PCA. The study objective was to compare this novel Oral-PCA technology with the gold standard IV-PCA. The study hypothesis was that the PCOA® Acute modality for Oral-PCA is noninferior to the IV-PCA and could therefore replace it for postoperative pain management. To assess this hypothesis, we retrospectively selected medical records of patients who received the same medication (oxycodone) by PCA treatment after surgery. The study included 2 cohorts according to the PCA modality: the first cohort received oral oxycodone tablets via the PCOA® Acute device, while the second cohort received IV-oxycodone via an IV pump. Data extracted from selected medical records were analyzed to compare the two PCA modalities.

## 2. Methods

### 2.1. Study Design

A comparable, retrospective study was conducted at the Department of Anesthesiology, Intensive Care, Pain Medicine/Palliative Medicine, Center for Pain Medicine; Center for Weaning; GFO-Clinics Bonn/CURA Hospital, Bad Honnef in Germany. Due to the retrospective design of the study, the approval of the centralized Institutional Review Board (IRB) was not necessary.

Medical records of patients after the following surgeries were collected: gynecology: hysterectomy, orthopedic: hip endoprosthesis/hip surgery, osteosynthesis/extremity surgery, plate osteosynthesis, and spine surgeries.

The following two cohorts were selected for retrospective analysis:

  Test cohort: 44 medical records of inpatients who received postoperative Oral-oxycodone-PCA via the PCoA® Acute device, during the year 2019. Before surgery, patients signed consent to receive Oral-PCA through the device and had a short training session to demonstrate pill suction by the device's mouthpiece.

  Control cohort: 61 medical records of inpatients who received postoperative IV-oxycodone-PCA, during the year 2018.

Data of the two cohorts were collected at 2 sequential years according to the use of the PCA mode in the study site: once PCoA® Acute was deployed in the department, all eligible patients were offered Oral-PCA by PCoA® Acute, and therefore, IV-PCA was used only by patients who were not able to or refused to use the Oral-PCA. To minimize the bias in the patient population between the cohorts, the selection of medical records for the IV-PCA cohort was from one year before PCoA® Acute was deployed.

### 2.2. Participants

The study population comprised males and females, aged > 18, who underwent surgery and received oxycodone as their postoperative analgesic using a PCA device. The following demographic parameters were collected from the medical records: age, gender, BMI, employment status, intervention surgery and PCA treatment duration, and hospitalization stay.

Inclusion criteria: the following medical records were selected for analysis:

(1) Males and females, aged > 18
(2) Following an operative procedure with at least 3 days of hospital stay
(3) Surgery with expected postoperative pain levels >4, gynecology or orthopedic surgery
  - Gynecology: hysterectomy
  - Orthopedic surgery: osteosynthesis/limb surgery
(4) The patient used PCA device for pain management (PCoA® Acute or IV pump)
(5) The patient signed consent for anonymized data extraction
(6) The patient received oxycodone as an analgesic
(7) The patient received standard treatment for background pain medication (etoricoxib, dipyrone, and in case of contraindications, acetaminophen)

Exclusion criteria: the following medical records were excluded from screening:

1. Incomplete medical data
2. Incomplete or missing patient consent for data extraction
3. Medication diverging from the standard medication or medication that did not match the medication that was evaluated in the study (oxycodone)

Medication for postoperative pain: postoperative pain treatment is implemented as a standard operating procedure in the medical center: in both patient cohorts, basic pain medication consisting of oral nonopioid analgesics was given simultaneously with the PCA. All participants in both cohorts received the same postoperative pain medication.

Pain treatment started in the recovery room by titrating oxycodone intravenously before patients were given the PCA. After readmission to the ward, all patients received nonopioids: initially, dipyrone was prescribed in combination with etoricoxib to minimize opioid dosing.

Contraindications for the use of dipyrone were allergy and blood count abnormalities. Contraindications for etoricoxib were allergy, cardiovascular diseases, and renal impairment. The daily doses for these analgesics were standardized as follows: dipyrone: 4 × 1 g; etoricoxib: depending on weight, either 1 × 60 or 90 mg.

For opioid treatment, oral oxycodone was used. In addition to the “basic” analgesic treatment, controlled-release formulation of oxycodone/naloxone was used at daily doses of 2 × 5/2.5 mg when pain levels > NRS 4 were expected. One single opioid dose was used here for standardization. Basic opioid dose adjustment was based on individual factors, such as BMI and/or pain levels.

### 2.3. Intervention

Patients of both cohorts received intravenous oxycodone immediately after surgery in the recovery room. Once patients reported sufficient pain control with an NRS > 4, PCA devices PCoA® Acute or IV-PCA pump were applied.

The test cohort—Oral-PCA by PCoA® Acute: PCoA® Acute provides Oral-PCA at the bedside. The medication was a short-lasting formulation of oxycodone. The bolus dose was 5 mg, and the lockout interval was 2 hours. A maximum of 4 tablets were allowed within 8-hour dosing intervals, on patient's demand. Device setup and medication dispensation were performed as described previously [18]. (18)

The control group—IV-PCA by an IV pump: the electronic CADD® pump (Smiths Medical) was used for IV-PCA. The IV-PCA pump administered oxycodone intravenously. The bolus dose was 1 mg oxycodone with a lockout interval of 30 minutes.

### 2.4. Outcome Measures

Data collection was performed during the PCA treatment, from the PCA initiation at the recovery room (baseline) until its discontinuation at the ward. The assessment of outcome measures was continued twice daily until PCA treatment discontinuation.

The following outcome measures were used to compare between the two PCA modalities:

(a) Patients' demographic and treatment characteristics: the following data are recorded for each patient in the Acute Pain Service form: age, gender, BMI, employment status, surgery type, hospitalization duration, and PCA intervention duration;
(b) Reduction in pain score: absolute pain scores were assessed by the NRS scale (0 = no pain; 10 = worst pain imaginable). NRS assessment demonstrates reduction in the NRS score at rest and in movement during the PCA treatment. NRS scores were manually recorded by physicians and/or nurses twice a day during the PCA treatment, in the morning (8:00–9:00 AM) and in the evening (7:00–9:00 PM).
(c) Side effects related to the PCA treatment: side effects' data were manually recorded by physicians and/or nurses of the Department of Anesthesiology following patients' report and by completion of the obligatory standard form of the Acute Pain Service at the hospital. This is a questionnaire form containing a list of side effects, including nausea/emesis, sedation scores, and mobility scores. This form is part of the electronical patients' records available at the hospital database and is used for quality control and for clinical evaluations.
(d) Total oxycodone bolus dose administered: it is the actual total dose that was administered to the patient during the PCA treatment.
    Total oxycodone bolus dose requested by patients during lockout intervals: it is the total analgesic dose that was requested by the patients during the lockout intervals. These boluses were not actually administered to the patient, so as to prevent overdose. High dose demand during lockout indicates suboptimal pain control during the PCA and dissatisfaction from the pain treatment. The total bolus doses administered and requested were recorded by using the PCA devices (both the IV-pump and the PCoA® Acute). All doses of oxycodone bolus administrations, as well as the requested doses that were not administered, were documented by physicians and/or nurses of the Department of Anesthesiology, using the standard form of the Acute Pain Service. Records are listed in mg units. Additionally, all prescriptions of the actual given doses were documented in the electronical patient records. All these records are obligatory in the medical site. By doing so, potential discrepancies between prescriptions and administered analgesics can be detected.
(e) Difficulties experienced with the PCA modality used: data on technical difficulties were manually recorded by the nurse following patients' reports. Technical difficulties included usability errors, operational difficulties, and device malfunctions. All difficulties and malfunctions were documented in the Acute Pain Service form.

### 2.5. Sample Size

The test cohort (Oral-PCA) contains medical records of 44 patients. The control cohort contains medical records of 61 patients (IV-PCA). All data were analyzed based on the following rationale.

When the sample size in each group is 45, a two-group large-sample normal approximation test of proportions with a one-sided 0.05 significance level will have 80% power to reject the null hypothesis that the test and the standard are not equivalent (the difference in proportions, *p*_*T*_ − *p*_*S*_, is 0.10 or farther from zero in the same direction) in favor of the alternative hypothesis that the proportions in the two groups are equivalent, assuming that the expected difference in proportions is 0.0 and the proportion in the standard group is 0.95 [19].

### 2.6. Statistical Analysis

All measured variables and derived parameters were tabulated by descriptive statistics. For categorical variables, summary tables were provided giving sample size and absolute and relative frequency. For continuous variables, summary tables were provided giving sample size, arithmetic mean, standard deviation, median, minimum, and maximum.

Chi-square test was applied for testing the statistical significance of the difference between the study cohorts in the following categorical variables: surgery type, gender, employment status, and side effects.

The two-sample *T*-test was applied for testing the statistical significance of the differences between the study cohorts in the following continuous variables: age, BMI, hospitalization duration, NRS in rest and movement, and bolus dose given and required.

The chi-square and the *T*-test were used to compare baseline scores for both conditions.

The level of significance was *P* value = 0.05.

## 3. Results

### 3.1. Patient Disposition

**Figure 2:**
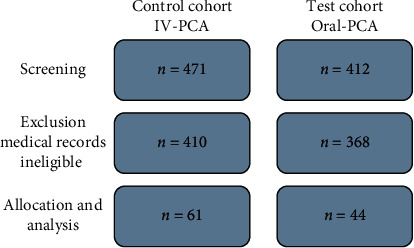
Patient disposition flow diagram.

The study was conducted at the Department of Anesthesiology, Interdisciplinary Intensive Care, Pain Medicine/Palliative Medicine, Center for Pain Medicine; Center for Weaning; GFO-Clinics Bonn/CURA Hospital, Bad Honnef. The patients' disposition flow diagram is illustrated in Figure 2. For the control cohort, medical records were screened of 471 inpatients, who underwent elective and trauma surgeries during the year 2018. 61 patients who used IV-PCA to receive oxycodone for the treatment of postoperative acute pain were found eligible to be included in the study. For the test cohort, medical records were screened of 412 patients who underwent elective surgery during the year 2019, out of whom 44 patients who used PCoA® Acute to receive oxycodone for the treatment of postoperative acute pain were found eligible to be included in the study.

The reasons for the exclusion of medical records during the screening were as follows: (1) other surgery types that were not included in the inclusion criteria, such as spine surgery or other abdominal gynaecological interventions; (2) use of analgesics other than oxycodone or use of nonopioids; (3) missing data due to incomplete forms or documented refusal by the patient for data extraction.

Medical records were collected for patients with two main types of surgery intervention: gynecology (hysterectomy) and orthopedic (hip endoprosthesis/hip surgery, osteosynthesis/extremity surgery). Patients' distribution was comparable between the two surgery types.

The data analysis of the study variables is shown for the entire study population (all-patients group).  Table 1 demonstrates the distribution of surgeries in the study population. The chi-square analysis demonstrates that there is a comparable patient distribution between surgery types (*P* = 0.084, chi-square = 2.9927).

Tables 2 and 3 demonstrate baseline variables of patients participating in the study. The mean age was comparable between cohorts (59.89 and 55.14 years in cohorts IV-PCA and Oral-PCA, respectively, *P* = 0.078), as well as the BMI (25.72 and 26.79 kg/m^2^ in cohorts IV-PCA and Oral-PCA, respectively, *P* = 0.260) and the PCA intervention duration (2.3 and 2.36 days in cohorts IV-PCA and Oral-PCA, respectively, *P* = 0.650). Gender distribution was also comparable between cohorts with more than 80% of patients being female due to a high number of gynecology interventions, *P* = 0.638. However, employment status was different between cohorts with 68.2% and 45.9% employed patients in the Oral-PCA and IV-PCA cohorts, respectively (*P* = 0.024).

Mean total hospitalization duration was 7.54 and 5.59 days in cohorts IV-PCA and Oral-PCA, respectively (*P* = 0.01), indicating longer hospitalization.

Notably, for the gynecology subgroup, all variables were comparable between cohorts. However, in the orthopedic subgroup, significant differences were found between cohorts in the following patients' variables: mean age, employment status, and hospitalization duration. The orthopedic subgroup of the IV-PCA cohort included older patients (mean age is 68.3 years), with an unemployment rate of 82.4% and mean hospitalization duration of 9.97 days (data not shown). All these variables are significantly higher compared to the data available for the all-study group.

### 3.2. Outcome Measures

#### 3.2.1. Pain Score Reduction

The NRS (Numerical Rating Scale) pain score rates pain levels at rest and during movement [1]. NRS scoring was recorded twice daily since the PCA initiation (initial NRS) until the day of its discontinuation (end NRS). Notably, the PCoA® Acute device enables ongoing NRS recording by the patient on the device touchscreen, after each dose taken. However, to enable a comparison of rest and movement NRS between cohorts, we compared the initial and the end NRS values as rated by the patients and recorded manually by the nurse.  Table 2 demonstrates the NRS scores at rest and movement, as well as the NRS reduction, from PCA initiation to termination in both cohorts.

A comparison of NRS data between the IV-PCA and the Oral-PCA cohorts revealed that there is no significant difference between cohorts in the mean initial NRS score in both rest and movement. The initial NRS scores at rest were 3.26 and 3.77 (*P* = 0.235), and the initial NRS scores in movement were 5.74 and 5.66 in the IV-PCA and Oral-PCA cohorts, respectively (*P* = 0.848). This result indicates that the initial pain level was comparable between the cohorts.

End NRS scores at rest were also comparable between the cohorts (1.66 and 1.5 for IV-PCA and Oral-PCA cohorts, respectively, *P* = 0.59). Interestingly, the difference in the end NRS in movement was significant between cohorts; the end NRS score in movement was 3.69 in the IV-PCA cohort and only 2.82 in the Oral-PCA cohort (*P* = 0.027).

Table 2 also demonstrates the NRS reduction at rest and in movement, following PCA treatment. Results show that the NRS reduction is higher in the Oral-PCA cohort compared with the IV-PCA cohort. Rest NRS reduction was −1.61 and −2.27 in the IV-PCA and Oral-PCA, respectively (*P* = 0.077). This trend was more significant for movement NRS, which was −2.05 and −2.84 in the IV-PCA and Oral-PCA, respectively (*P* = 0.039). These results indicate that Oral-PCA may be more effective than IV-PCA in movement pain reduction after surgery.

#### 3.2.2. Side Effects

Side effects related to the pain treatment were reported by patients and manually recorded by the nurse during the PCA treatment. The recorded side effects for the all-patients group are listed in Table 4.

Table 4 demonstrates that most of the study participants did not experience side effects related to the pain treatment; only 14.8% of patients in the IV-PCA cohort and 25% of patients in the Oral-PCA cohort reported side effects (*P* = 0.187, chi-square = 1.7403). These results indicate that both cohorts are comparable in inducing side effects. The most reported side effect was vomiting (9.8% of the IV-PCA patients and 20.5% of the Oral-PCA patients). Nausea was reported only by patients receiving Oral-PCA (22.7%), while dizziness and fatigue were reported only by patients receiving IV-PCA (6.6% and 3.3%, respectively).

#### 3.2.3. Treatment Boluses Administered and Requested during Lockout Intervals

**Figure 3:**
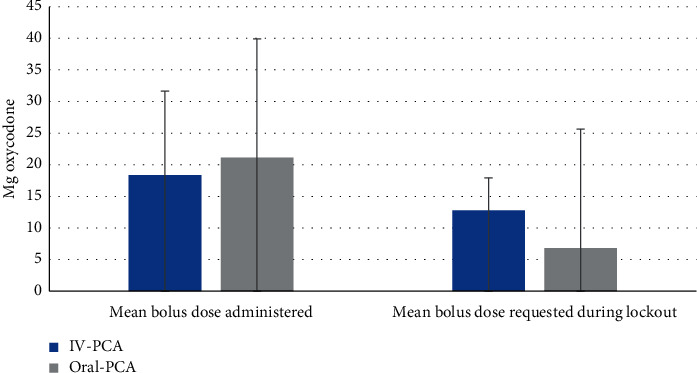
Comparison of mean total bolus dose administered and requested during lockout intervals, during the PCA treatment, in both cohorts, by means of *T*-test for independent samples.

Figure 3demonstrates the mean total bolus dose administered to patients and requested by patients during lockout intervals during the PCA treatment in both cohorts. Notably, the bolus dose and regimen were different between cohorts (during IV-PCA, patients received bolus doses of 1 mg oxycodone, with a lockout interval of 30 minutes. During Oral-PCA, patients received bolus doses of 5 mg oxycodone with a lockout interval of 2 hours). It was found that the mean total dose administered was comparable between cohorts (23 mg and 17.35 mg in the IV-PCA and Oral-PCA cohort, respectively, *P* = 0.27). However, the mean total dose requested during lockout was significantly different between the cohorts (12.8 and 6.82 mg in the IV-PCA and Oral-PCA cohort, respectively, *P* = 0.004, *T*-test).

#### 3.2.4. Device Malfunction and Difficulties Experienced with the PCA Modality Used

Difficulties encountered when using the PCA devices were recorded during treatment.  Table 5 demonstrates that difficulties with the PCA modality were comparable between cohorts (*P* = 0.056, chi-square = 3.6418). However, 19.7% of patients receiving IV-PCA and 36.4% of patients receiving Oral-PCA reported difficulties: 9.8% of patients in the IV-PCA cohort reported dislocation of the peripheral intravenous catheter (6 patients) and 8.2% of patients reported practice difficulties or a high need for information (5 patients). In contrast, only 1 patient (2.3%) in the Oral-PCA cohort reported practice difficulties. 18.2% of the Oral-PCA cohort (8 patients) reported difficulties in drug administration using the suction. 6.8% of them (3 patients) reported system errors. The Oral-PCA by PCOA® Acute is a new technology, and therefore, more operational difficulties were encountered during treatment. Notably, operation errors were corrected, and usability was improved with time.

## 4. Discussion

This study aimed to evaluate a new Oral-PCA modality, PCoA® Acute, by comparing its effectiveness and usability to the gold standard IV-PCA modality. The study question was whether both PCA modalities are comparable for postoperative PCA management. A positive answer may suggest that the IV-PCA can be replaced by this Oral-PCA technology in order to overcome its inherent substantial limitations, including invasiveness, mobility limitation, high cost, and labor intensity.

The study results demonstrate that Oral-PCA by PCoA® Acute is noninferior to the IV-PCA in terms of pain control capability and usability. Moreover, the Oral-PCA was found to be superior to the IV-PCA in movement pain reduction. These results suggest that this Oral-PCA technology can potentially replace the IV-PCA at least for the tested clinical cases (gynecology and orthopedic surgeries) and for the analgesic used (oxycodone).

The study design was retrospective; medical records included were those of patients after hysterectomy and orthopedic surgeries, who received oxycodone as the PCA medication and standard background pain medication. A retrospective study design is commonly used to evaluate PCA treatment [20, 21]. To overcome potential bias arising from the retrospective design, medical records of the IV-PCA cohort were selected from a time period preceding the deployment of PCoA® Acute in the department.

Most of the baseline variables were comparable between cohorts: mean age (*P* = 0.078), BMI (*P* = 0.26), PCA intervention duration (*P* = 0.65), and gender (*P* = 0.638). However, some variables were significantly different between cohorts: employment status (68.2% vs. 45.9% in the Oral-PCA and IV-PCA cohort, respectively, *P* = 0.024) and hospitalization duration (5.59 vs. 7.54 days in the Oral-PCA and IV-PCA cohort, respectively, *P* = 0.01). These differences between cohorts are explained by the high number of hip surgeries: 47% in the IV-cohort vs. 23.5% in the Oral-PCA cohort. Hip surgeries usually required at older age, which is correlated with unemployment (retirement) and longer hospitalization duration. It is possible that the Oral-PCA mode was offered to younger patients with less complicated surgeries since this is a new technology which requires patients' engagement and intact cognition. However, IV-PCA requires patients with similar characteristics. It is therefore questionable whether the surgery type had any impact on the effectiveness of the PCA technique.

Measuring pain reduction by changes of subjectively assessed NRS scores is commonly used to evaluate the effectiveness of the pain treatment [22]. Our results demonstrate that there is no significant difference between cohorts in initial NRS at rest (*P* = 0.235) and in movement (*P* = 0.848), indicating that both PCA modes were initiated with comparable pain levels.

No significant differences between cohorts were found in NRS reduction at rest, although it was higher in the Oral-PCA cohort compared with the IV-PCA cohort (2.27 vs. 1.61, respectively, *P* = 0.077). This may be explained by the consequently applied background analgesics resulting in sufficient pain control at rest. However, NRS reduction in movement was significantly higher in the Oral-PCA cohort compared with the IV-PCA cohort (2.84 vs. 2.05, respectively, *P* = 0.039). This can be explained by the higher bolus dose provided by the Oral-PCA. For Oral-PCA, the minimal bolus dose is 5 mg oxycodone, while for IV-PCA, the maximal safe bolus dose is 1 mg oxycodone. This difference may explain the better pain reduction obtained by using the Oral modality. Better mobility was also found with other noninvasive PCA modalities [23] such as the SSTS [12] and FITS when compared to the IV-PCA [13].

A recent study compared oral oxycodone administration with IV-oxycodone-PCA for patients after cesarean section. Results showed no differences in NRS pain scores or satisfaction between the study cohorts. Postoperative recovery and satisfaction with pain treatment were similar in both cohorts, and both methods were well tolerated [24]. This study supports our finding that Oral-PCA is noninferior to IV-PCA in postoperative pain reduction.

Side effects are a critical parameter in the evaluation of pain treatment. 75% of the patients in the Oral-PCA cohort and 85.2% of the patients in the IV-PCA cohort did not experience side effects (*P* = 0.187). In the IV-PCA cohort, patients reported more dizziness and fatigue, despite the low bolus dose. In the Oral-PCA cohort, patients mainly reported nausea and vomiting, probably due to the high bolus dose administered orally. These findings may shed light on the effect of opioid pharmacokinetics on the emergence of side effects and should be further investigated by a relevant study design.

The cumulative oxycodone dose administered to patients during the PCA treatment was comparable between cohorts (*P* = 0.27). Oral oxycodone was demonstrated previously as a noninferior treatment to the IV-PCA in pain relief [25] and may offer some logistics and cost advantages [26]. In our department, oxycodone was used, but there are no limitations as to the use of other oral opioids, such as oral morphine, hydromorphone, and tapentadol.

The total oxycodone dose requested by patients during lockout intervals was different between cohorts. The mean dose requested was lower in the Oral-PCA cohort compared with the IV-PCA cohort (6.82 mg vs. 12.8 mg, respectively, *P* = 0.004). One explanation may be that the PCoA® Acute device shows the availability of the next pill on its touchscreen, while this information is not shown to the IV-PCA user. Another explanation is the higher oral bolus dose administered, providing better pain control compared to the IV-PCA.

Since this was a retrospective study, the usability and ease of use of the PCA modalities were not evaluated directly. However, data were collected regarding technological difficulties experienced by patients. Notably, PCoA® Acute is a new technology for physicians, nurses, and patients alike, and the minimal experience may be a contributing factor in the technical difficulties encountered. However, the difference between cohorts was not significant (*P* = 0.056). 15.9% of the patients in the Oral-PCA cohort encountered operational difficulties or errors, which have since then been fixed. 18.2% of the patients reported difficulties in the pill administration mode. Suction of a solid pill is a novel approach and requires acceptance as well as a certain level of training and skill. It was previously shown that following such training and gaining of experience, patients improved their confidence, and 90% reported high ease of use and satisfaction [18]. 9.8% of the patients in the IV-PCA cohorts encountered device-related difficulties, and 8.2% of the patients reported practice difficulties. These results indicate that PCoA® Acute is noninferior to the IV pump in terms of technical difficulties.

Future strategy: following our real-world experience with the PCoA® Acute device, the following limitations were identified:

1. The device is limited to specific types of analgesic tablets and can be used only with one type of tablet per treatment
2. The device is large, fixed to the patient's bedside, and is restricted to the hospital setting
3. Lacking an internet platform, therapy data cannot be presented online

**Figure 4:**
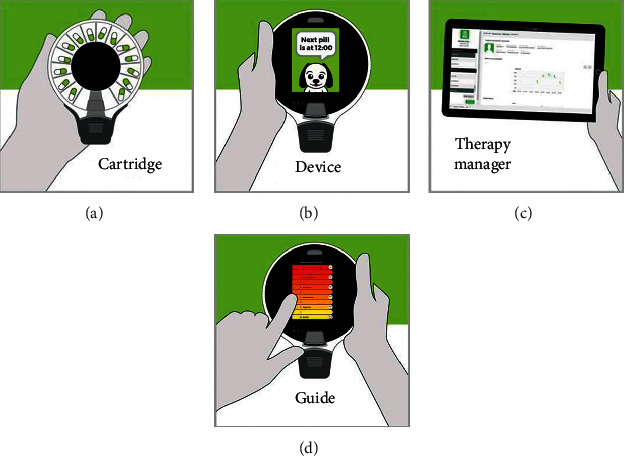
The ReX platform components.

To overcome these limitations, we developed ReX [27], a handheld, mobile, and flexible medication management platform, which comprises a dispenser device, disposable pill cartridges, a digital guide, and an online Therapy Manager serving also as a database (Figure 4).

Both ReX and the PCoA® Acute device are based on the innovative technology of tracking and dispensing solid pills into the patient's mouth. ReX demonstrates the following benefits:

  Pills are supplied in preloaded, disposable, tamper-proof cartridges. This enables flexibility in pill type, dose, and quantity.
  ReX is small and mobile and connected to an online application (the Therapy Manager). Hence, it allows monitoring of patients in their homes after being discharged from the hospital or outpatients taking pain medication.

We aim to implement ReX, as an improved version of PCoA® Acute, for pain medication management at the hospital and in the home setting.

## 5. Study Limitations

This study's limitations stem from its retrospective design: the lack of patients' randomization generated unbalanced cohorts with differences in patients' variables. To overcome this limitation, careful screening of medical records was conducted to enable comparable study cohorts.

The study protocol was not registered due to the retrospective design and the fact that it was a noninterventional study.

Bolus doses varied due to the unavailability of low-dose tablets of oral oxycodone, while higher IV doses could not be used due to severe adverse events. Different opioid dosages are correlated with pharmacokinetics affecting opioid-related side effects, pain scores, and boluses' request by the patients.

Patient-reported pain scores in movement were not assessed for the mobility level, e.g., walking distance capability. Such assessment could support the data indicating that Oral-PCA improves postoperative mobility.

A prospective randomized controlled (RCT) study design for the evaluation of Oral-PCA in comparison to IV-PCA can provide further insights by circumventing the influence of age, surgery type, and analgesic medication.

Another limitation of this study relates to data availability; side effects and technical difficulties were recorded during the nurse's routine visit, rather than by systematic evaluation of patients' condition and satisfaction during their hospitalization through validated questionnaires and technological means. A well-designed RCT could overcome this limitation as well.

## 6. Conclusions

This retrospective study indicates that Oral-PCA by PCoA® Acute provides pain control and usability which is noninferior to that achieved by the IV-PCA for postoperative analgesia after hysterectomy and limb surgeries.

Reduction in NRS scores at rest was comparable between the PCA modes, whereas patients' mobility was significantly better in the Oral-PCA cohort. Both modes were well tolerated with similar rates of pain-related side effects and comparable rate of technical difficulties. The results demonstrate the potential of our Oral-PCA technology to replace the IV-PCA for various patient populations and support the hypothesis that Oral-PCA provides an effective pain treatment which is noninvasive and can improve patients' postoperative mobility while reducing cost and labor of the clinical team.

## Figures and Tables

**Table 1:** Comparison of surgery type across study cohorts by means of a chi-square test.

	ControlIV-PCA		TestOral-PCoA		All		*P*value (chi-square)
	*N*	%	*N*	%	*N*	%	
Surgery intervention							0.084
Gynecology-hysterectomy	27	44.3	27	61.4	54	51.4	
Orthopedics	34	55.7	17	38.6	51	48.6	
Hip endoprosthesis/hip surgery	16	47.1	4	23.5	20	39.2	
Osteosynthesis/extremity surgery	18	52.9	13	76.5	31	60.8	

**Table 2:** Cross-cohort comparison of patients' continuous variables by *T*-test for independent samples.

	Control-IV-PCA					Test-Oral-PCA					*P* value (*T*-test)
	*N*	Mean	Std	Min	Max	*N*	Mean	Std	Min	Max	
Age (years)	61	59.89	16.14	21.00	91.00	44	55.14	11.2	23.00	87.00	0.078
BMI (kg/m^2^)	61	25.72	4.20	16.70	34.30	44	26.79	5.51	18.80	44.80	0.260
PCA intervention (days)	61	2.30	0.59	1.00	4.00	44	2.36	0.87	1.00	4.00	0.650
Hospitalization duration (days)	61	7.54	4.54	3.00	22.00	44	5.59	2.35	2.00	15.00	0.01

Initial NRS at rest	61	3.26	1.94	0.00	8.00	44	3.77	2.44	0.00	10.00	0.235
Initial NRS in movement	61	5.74	2.00	1.00	10.00	44	5.66	2.18	0.00	10.00	0.848
End NRS at rest	61	1.66	1.44	0.00	7.00	44	1.50	1.50	0.00	7.00	0.59
End NRS in movement	61	3.69	2.08	0.00	10.00	44	2.82	1.79	0.00	6.00	0.027
NRS score reduction in rest	61	−1.61	1.78	−8.00	3.00	44	−2.27	2.03	−8.00	1.00	0.077
NRS score reduction in movement	61	−2.05	1.88	−6.00	4.00	44	−2.84	1.96	−8.00	1.00	0.039

**Table 3:** Cross-cohort comparison of baseline categorical variables by chi-square for independent samples.

	ControlIV-PCA		TestOral-PCoA		All		*P* value (chi-square)
	*N*	%	*N*	%	*N*	%	
*Gender*							0.638
Female	52	85.2	36	81.8	88	83.8	
Male	9	14.8	8	18.2	17	16.2	

*Employment status*							0.024
Employed	28	45.9	30	68.2	58	55.2	
Unemployed	33	54.1	14	31.8	47	44.8	

**Table 4:** Rate and specifications of side effects related to pain treatment in the general study population. Statistical analysis was performed by the chi-square method.

	IV-PCA		Oral-PCA		*P* value (chi-square)
	*N*	%	*N*	%	
*Side effects (yes/no)*					0.187
No	56	85.2	33	75.0	
Yes	9	14.8	11	25	

*Side effects' specifications*					
Dizziness	4	6.6	0	0	
Fatigue	2	3.3	0	0	
Nausea	0	0	10	22.7	
Vomiting	6	9.8	9	20.5	

**Table 5:** Comparison of rate and specifications of difficulties with the PCA modalities by means of chi-square for independent samples.

	IV-PCA		PCoA		*P* (chi-square)
	*N*	%	*N*	%	
*Difficulties experienced with the PCA modality used (yes/no)*					0.056
No	49	80.3	28	63.6	
Yes	12	19.7	16	36.4	

*Difficulties with the PCA modality used (specifications)*					
Difficulty of the administration mode (suction and others)	0	0	8	18.2	
Difficulty or dislocation of the peripheral intravenous catheter	6	9.8	0	0	
Error (device and system)	0	0	4	9.1	
Operation difficulties	0	0	3	6.8	
Practice difficulties, high need for information	5	8.2	1	2.3	
Subjectively insufficient analgesia, the patient prefers oral medication	1	1.6	0	0	

## Data Availability

The data related to this study are available from the corresponding author upon request (ronit.s@dosentrx.com).

## Abbreviations

FITS: Fentanyl iontophoretic transdermal system
PCA: Patient-controlled analgesia
IV: Intravenous
PCoA: Patient-controlled oral analgesics
PO: Postoperative
Std: Standard deviation
NRS: Numerical Rating Scale
RCT: Randomized controlled trial
RFID: Radio-frequency identification
SSTS: Sufentanil sublingual tablet system.
